# Circulating microRNA Profile throughout the Menstrual Cycle

**DOI:** 10.1371/journal.pone.0081166

**Published:** 2013-11-14

**Authors:** Kadri Rekker, Merli Saare, Anne Mari Roost, Andres Salumets, Maire Peters

**Affiliations:** 1 Competence Centre on Reproductive Medicine and Biology, Tartu, Estonia; 2 Department of Obstetrics and Gynecology, University of Tartu, Tartu, Estonia; 3 Institute of Biomedicine and Translational Medicine, University of Tartu, Tartu, Estonia; John Hopkins University School of Medicine, United States of America

## Abstract

Normal physiological variables, such as age and gender, contribute to alterations in circulating microRNA (miRNA) expression levels. The changes in the female body during the menstrual cycle can also be reflected in plasma miRNA expression levels. Therefore, this study aimed to determine the plasma miRNA profile of healthy women during the menstrual cycle and to assess which circulating miRNAs are derived from blood cells. The plasma miRNA expression profiles in nine healthy women were determined by quantitative real time PCR using Exiqon Human Panel I assays from four time-points of the menstrual cycle. This platform was also used for studying miRNAs from pooled whole blood RNA samples at the same four time-points. Our results indicated that circulating miRNA expression levels in healthy women were not significantly altered by the processes occurring during the menstrual cycle. No significant differences in plasma miRNA expression levels were observed between the menstrual cycle time-points, but the number of detected miRNAs showed considerable variation among the studied individuals. miRNA analysis from whole blood samples revealed that majority of miRNAs in plasma are derived from blood cells. The most abundant miRNA in plasma and blood was hsa-miR-451a, but a number of miRNAs were only detected in one or the other sample type. In conclusion, our data suggest that the changes in the female body during the menstrual cycle do not affect the expression of circulating miRNAs at measurable levels.

## Introduction

MicroRNAs (miRNAs) are a class of small non-coding RNAs that regulate gene expression at the post-transcriptional level. miRNAs have been found in all mammalian tissues and cell types examined so far and play important roles in a variety of physiological and pathological processes [1]. In addition to cells and tissues, miRNAs are present in extracellular body fluids such as plasma, serum, urine, and saliva [2–4]. Although the origin of cell-free miRNAs in circulation is not entirely clear, miRNAs are believed to be released to the extracellular space from damaged or apoptotic cells and are also actively secreted by cells via exosomes or exocytosis [5,6].

Despite high RNase concentration in plasma and serum, circulating miRNAs are relatively stable [7] and therefore considered as good candidates for biomarker studies. Several studies have shown that the expression pattern of circulating miRNAs can be used to identify patients with cancer, cardiovascular and intestinal diseases [8,9]. In addition to these pathologies, circulating miRNAs are proposed to be a promising source of potential biomarkers for non-invasive diagnosis of infertility and gynaecological diseases [10], such as endometriosis [11,12] and ectopic pregnancy [13].

In addition to physiological factors, pre-analytical steps, including specimen collection and sample handling, can affect the plasma miRNA detection and quantification [14]. For example, haemolysis, a phenomenon occurring during sample collection, can increase red blood cell derived miRNA concentration in plasma [15,16]. Blood cells are major contributors to circulating cell-free miRNAs and plasma miRNA levels are directly influenced by blood cell counts [16]. Most cancer-specific circulating miRNA biomarkers reported so far are highly expressed in blood cells and the alterations in blood cell counts may have an impact on the biomarker specificity, as the changes observed may reflect the sample characteristics rather than the disease [16]. Accordingly, it is important to find circulating miRNA biomarkers that are not highly expressed in blood cells. 

The female body undergoes many physiological and hormonal changes during the menstrual cycle with major alterations occurring in the endometrium. Several studies have shown fluctuations in endometrial miRNA profiles during the menstrual cycle [17–20]. For instance, Kuokkanen et al. (2010) reported differential miRNA expression patterns in endometrial epithelial cells during the late proliferative and mid-secretory menstrual phases, suggesting that some miRNAs are hormonally regulated in the human endometrium. These results refer that miRNAs in the endometrium play an important role in gene expression regulation throughout the menstrual cycle. Cyclic changes in miRNA expression levels have also been observed in other tissues. A study conducted with sheep ovarian tissues demonstrated differential miRNA expression in follicles and corpora lutea during the ovine oestrous cycle [21]. Based on this, it is reasonable to assume that the changes in miRNA profile occurring in the female body during the menstrual cycle may be reflected in plasma miRNA expression levels as well.

Therefore, before miRNAs can be used as non-invasive diagnostic tools in women, it is obligatory to know how the plasma miRNA expression profile fluctuates during the menstrual cycle in healthy women of reproductive age. Since circulating miRNAs have not been investigated in relation to the menstrual cycle, the main aim of the current study was to determine the plasma miRNA profile during the menstrual cycle of healthy women. Secondly, in order to find out which plasma miRNAs are derived from blood cells, miRNAs were studied along the menstrual cycle in whole blood samples of healthy individuals. 

## Materials and Methods

### Ethics statement

The study was approved by the Ethics Review Committee on Human Research of the University of Tartu (Tartu, Estonia) and written informed consent was obtained from all participants.

### Study participants

A total of 12 healthy young adult women (mean age ± SD, 25.7 ± 4.8 years; body mass index 22.0 ± 3.2 kg/m^2^) were enrolled in this study. A questionnaire was administered to obtain information regarding general health characteristics, including menstrual anamnesis, use of medications, presence of systemic diseases and other health conditions. Study subjects defined themselves as healthy; none of the studied individuals suffered from gynaecological, inflammatory or any chronic diseases at the time of participation. No previous history of endometriosis or autoimmune disorders was reported. None of the recruited participants had used hormonal medications for at least 3 months prior the study. All the women reported regular menstrual periods with intervals up to 35 days. The duration of menstruation was within 3 to 7 days. Three participants out of 12 were excluded from the study – two women due to illness during the sampling period, and the luteinizing hormone (LH) levels in plasma of one individual did not correspond to the presumed menstrual cycle time-points. The final number of participants included in the study was 9.

### Sample preparation and RNA isolation

Peripheral blood samples from healthy women at four time-points during one menstrual cycle were collected into 9 ml EDTA tubes and were processed within an hour. The first time-point for sample collection was the first day of the menstruation (cycle day 1); the second time-point was the seventh day from the beginning of the menstruation (cycle day 7); the third time-point was determined as the time of the LH surge (LH day 0) and the fourth time-point was seven days after the LH surge (LH day 7). The time of the LH surge was assessed with urine-based LH test Baby Time™ (Zer Hitech Ltd., Israel); blood samples from LH day 0 were collected at the same day the LH surge was detected. In order to confirm the results of the urine-based LH test, LH levels were further measured from plasma samples at cycle day 7 and LH day 0. The average LH values for cycle day 7 and LH day 0 were 4.9 ± 1.4 U/L (mean ± S.E.M.) and 28.4 ± 11.1 U/L, respectively. Blood samples were centrifuged at 1600 *g* for 10 min to separate plasma from the blood cells. Plasma was further processed by an additional centrifugation step at 16 000 *g* for 10 min. All the centrifugation steps were performed at 4°C. Buffy coat and plasma fractions were collected into separate tubes. Red blood cell (RBC) lysis was not performed on buffy coat samples (from further on referred as “whole blood samples”). No signs of haemolysis could be detected in plasma by visual investigation. Also, NanoDrop 2000 (Thermo Scientific, Wilmington, USA) spectrophotometer measurements from wavelengths λ200-800 nm did not show a distinct peak at 414 nm corresponding to maximum absorbance of oxy-haemoglobin in plasma [15]. Three volumes of Trizol LS Reagent (Invitrogen, Life Technologies, USA) were added to plasma and blood for RNA isolation; samples were stored at −80°C until further processing. RNA was isolated from 4 ml of plasma or 250 µl of blood using miRNeasy Mini kit (Qiagen, Hilden, Germany) according to the manufacturer’s instructions with minor modifications to the protocol where Trizol LS Reagent was used instead of QIAzol Lysis Reagent. A final elution volume for RNA isolation was 50 µl. 

Quality and quantity of RNA isolated from whole blood samples were assessed with RNA 6000 Nano chips (Agilent Technologies, Palo Alto, CA, USA) and all the RIN values were ≥8.0. Agilent RNA chips were not used for the quality assessment of plasma RNA samples as circulating cell-free RNA in plasma consists of short fragments and therefore ribosomal RNA peaks are not present. The presence of miRNAs in all RNA samples was assessed with Agilent Small RNA chips. 

### miRNA profiling from plasma and blood samples

Plasma samples from nine women collected at four time-points of the menstrual cycle were used for miRNA profiling (altogether 36 plasma samples). In addition, pooled whole blood RNA samples were used for miRNA profiling. Pools were generated from whole blood RNA samples isolated from six healthy individuals (six out of nine individuals from the plasma study) at four time-points of the menstrual cycle (four pools, one for every time-point). For each pool, RNAs were mixed at equal concentration (400 ng of total RNA). RNA was treated with DNase using TURBO DNA-free™ kit (Ambion, Austin, TX, USA) according to the manufacturer’s instructions. 

Reverse transcription reactions were performed using the Universal cDNA Synthesis Kit (Exiqon, Vedbaek, Denmark). For each reaction 4 µl of RNA corresponding to RNA amount isolated from 320 µl of plasma or 20 ng of total blood RNA was used. 

Exiqon miRCURY LNA microRNA Human panel I (V2.M) quantitative real time PCR (qRT-PCR) assays were used to determine the expression profile of 375 human miRNAs. Amplification was performed on the 7900HT thermocycler (Applied Biosystems, Foster City, CA, USA) using cycling parameters recommended by Exiqon. To determine the technical variation between the used Human Panel I plates the inter-plate calibrators (IPC) were analysed. IPC (UniSp3) levels were highly similar among the samples with Ct values of 19.6 ± 0.5 (mean ± SD) and 19.7 ± 0.2 for plasma and blood samples, respectively.

qRT-PCR data were analysed using the GenEx software 2.0 (MultiD Analyses, Sweden). Threshold cycle (Ct) values greater than 36 were considered to be below the detection level of the assay. qRT-PCR data set was normalised by global mean strategy [22]. Relative expression was calculated with the comparative Ct method [23]. Plasma miRNA expression levels between the menstrual cycle time-points were compared with one-factor ANOVA from relative miRNA expression values. Differences in miRNA expression between blood and plasma samples were determined by two-tailed unpaired t-test. P-values were adjusted for multiple testing according to the Bonferroni correction. Significance threshold was set to a fold change ≥ 2 with a corrected P-value ≤ 0.00026 (dataset with 199 detected miRNAs) or ≤ 0.00027 (dataset with 190 detected miRNAs) for the comparison of plasma miRNA expression between the time-points and for the comparison of blood and plasma miRNAs, respectively. A two-factor ANOVA (without replication) was performed on the number of detected plasma miRNAs to assess the differences between the menstrual cycle time-points and studied individuals. For this analysis, a P-value ≤ 0.05 was considered statistically significant.

## Results

### miRNAs expressed in plasma

Plasma samples from nine women at four different time-points during the menstrual cycle (altogether 36 samples) were profiled for 375 miRNAs using Exiqon real time PCR assays. On average, 197 out of 375 miRNAs (ranging from 94 to 315 miRNAs) were detected at the threshold level Ct < 36 from all plasma samples. Fifteen most abundant miRNAs detected in the plasma of healthy women are presented in Table 1. hsa-miR-1979, hsa-miR-720, hsa-miR-886-3p and hsa-miR-886-5p were also detected at high levels in plasma samples, but these sequences are no longer considered as miRNAs according to miRBase release 18 and were removed from the analysis.

**Table 1:** Most abundant miRNAs detected from plasma and blood samples of healthy women.

**miRNAs detected in plasma samples**	**miRNAs detected in blood samples**
hsa-miR-451a	hsa-miR-451a
hsa-miR-486-5p	hsa-miR-144-3p
hsa-miR-92a-3p	hsa-miR-16-5p
hsa-miR-320a	hsa-miR-15a-5p
hsa-let-7b-5p	hsa-miR-19b-3p
hsa-miR-93-5p	hsa-miR-142-3p
hsa-miR-223-3p	hsa-miR-486-5p
hsa-miR-185-5p	hsa-miR-92a-3p
hsa-miR-150-5p	hsa-miR-20a-5p
hsa-let-7d-3p	hsa-miR-223-3p
hsa-miR-25-3p	hsa-miR-103a-3p
hsa-miR-484	hsa-miR-93-5p
hsa-miR-16-5p	hsa-miR-106a-5p
hsa-miR-103a-3p	hsa-let-7g-5p
hsa-miR-106a-5p	hsa-let-7b-5p

miRNAs are named according to miRBase (release 18).

### miRNA profile during the menstrual cycle

The overall plasma miRNA expression profiles of healthy women were similar throughout the menstrual cycle. None of the miRNA expression differences reached the level of significance required for multiple testing. The hierarchical clustering analysis showed that the samples did not cluster according to the menstrual cycle time-point but a considerable clustering was formed by study subjects (Figure S1). 

In addition to miRNA expression levels, the number of detected miRNAs was compared between the study groups. The average number of detected miRNAs was similar (ranging from 192 to 202) among the studied menstrual cycle time-points (Table 2). The average number of detected miRNAs ranged from 135 to 289 if the data were grouped by study subjects (Table 2). The number of detected miRNAs showed no statistically significant differences between menstrual cycle time-points (P = 0.8), but the variation among the studied individuals was statistically significant (P = 1.5 × 10^-7^).

**Table 2:** The number of miRNAs detected from plasma samples with Exiqon assays.

	**Menstrual cycle time-point**				
**Study participant ID**	**Cycle day 1**	**Cycle day 7**	**LH day 0**	**LH day 7**	**Average number of miRNAs (mean ± SD)**
E10	221	175	199	202	**199 ± 19**
E11	175	230	242	252	**225 ± 34**
E12	172	141	151	179	**161 ± 18**
E13	115	162	113	148	**135 ± 24**
E15	169	148	174	172	**166 ± 12**
E16	186	229	214	239	**217 ± 23**
E17	255	241	233	257	**247 ± 11**
E35	265	315	311	263	**289 ± 28**
E37	173	94	180	101	**137 ± 46**
**Average number of miRNAs (mean ± SD)**	**192 ± 47**	**193 ± 67**	**202 ± 57**	**201 ± 56**	

SD – standard deviation

### miRNAs expressed in whole blood

To identify which miRNAs are expressed in blood cells, pooled RNA from whole blood samples of six individuals from each menstrual cycle sampling point was used. Data analysis of Exiqon assays showed that on average, 290 out of 375 miRNAs (ranging from 285 to 296 miRNAs) were detected at the threshold level of Ct < 36 from pooled samples. Fifteen most abundant miRNAs detected in the whole blood samples of healthy women are presented in Table 1. miRNA composition and levels were highly similar between the four pools (Table S1). 

### Comparison of miRNA expression in plasma and blood

**Figure 1:**
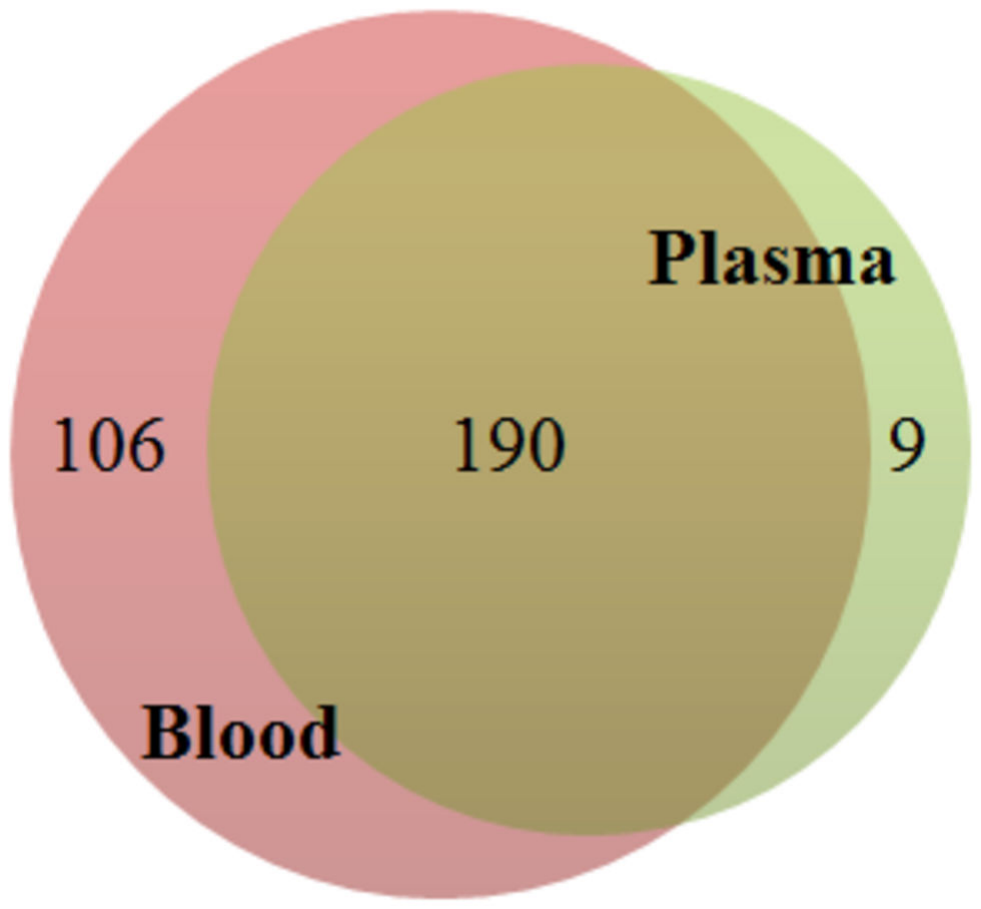
Venn diagram showing the number of common and unique miRNAs detected from different sample types.

In order to compare miRNA expression differences between plasma and blood, all the data from the Exiqon plates of the same sample type were used as technical replicates so that plasma samples, independent from the menstrual cycle day or the study subject, were compared to four pooled blood samples. For this analysis, all miRNAs below the threshold value Ct < 36 that were present in at least 50% of the samples in blood or plasma were considered as detected. From 375 miRNAs that were present on Exiqon plates 296 and 199 were detected from blood and plasma samples, respectively (Table S2). One hundred and ninety miRNAs were present in both fractions, 106 were uniquely detected in blood and nine in plasma samples (Figure 1; Table S2). Nine miRNAs (hsa-miR-451a, hsa-miR-486-5p, hsa-miR-92a-3p, hsa-let-7b, hsa-miR-93-5p, hsa-miR-223-3p, hsa-miR-106a-5p, hsa-miR-103a-3p and hsa-miR-16-5p) were represented among the 15 most abundant miRNAs in both sample types (Table 1). The most highly expressed miRNA in both sample types was hsa-miR-451a. In addition to nine miRNAs detected only in plasma, 41 miRNAs showed significantly higher and 40 miRNAs lower relative expression values in plasma compared to blood samples (fold change > 2; P < 0.00027 (Bonferroni correction); Table S3; Figure S1).

## Discussion

Recent studies have demonstrated that changes in global miRNA expression pattern in circulating body fluids and cells are influenced by several demographic and normal physiological variables. Age [24], gender [25] and ethnic origin [26] have been shown to be some of the factors contributing to miRNA expression level differences. In the present study, we investigated whether the changes occurring in the female body throughout the menstrual cycle influence plasma miRNA patterns of healthy young women. 

miRNAs regulate many cellular processes that also occur during the cyclic changes in the endometrium and other tissues. Several studies have demonstrated differential expression of miRNAs in endometrium during the menstrual cycle and have emphasised their role in endometrial differentiation into its receptive state [17,18,20]. For example, these studies reported up to seven fold differences in miRNA expression levels between receptive (LH day 7) and non-receptive (cycle day 12 or LH day 2) endometrium, where miR-30d, miR-30b, miR-31, miR-193a-5p, miR-203 showed up-regulation and miR-503 down-regulation in receptive endometrium [17,18,20]. McBride et al. (2012) observed nine miRNAs (miR-125a, miR-199a-3p, miR-125b, miR-99a, let-7c, miR-145, miR-31, miR-202 and miR-27b) with decreased expression and eight miRNAs (miR-503, miR-21, miR-29b, miR-142-3p, miR-34a, miR-152, miR-25 and miR-130a) with increased expression between the follicular and luteal stages in ovine ovarian tissues [21]. In our study, no differences in plasma miRNA expression levels between the menstrual cycle time-points were found. Based on this finding, we suggest that miRNA levels detectable in plasma of healthy women are not significantly altered by the processes occurring during the menstrual cycle. It is probable that miRNA expression changes in tissues are necessary for the local gene expression regulation but the fluctuations are insufficient to alter the miRNA patterns detectable in circulation. 

In addition to miRNA expression levels, we were interested in whether any differences in the numbers of detected miRNAs between the study groups can be observed. The number of detected miRNAs in our study showed significant inter-individual variation that exceeded the differences between the menstrual cycle time-points (Table 2). Thereby, it is likely that individual differences in the numbers of detected miRNAs may mask the possible fluctuations in miRNA profiles throughout the cycle. Healthy volunteers in our study were of the same age and ethnic origin; therefore it is unlikely that these factors had significant effect on miRNA levels.

Plasma miRNAs are a promising source of potential biomarkers for non-invasive diagnosis of many diseases. Most plasma miRNA biomarkers associated with different pathologies that have been discovered so far are also found to be expressed at high levels in blood cells. The alterations in blood cell counts and specimen handling procedures may have an impact on the candidate circulating cell-free miRNA biomarker specificity, as the changes observed may be caused by physiological changes rather than the disease [16]. Keeping in mind the latter, it is important to find circulating miRNA biomarkers that are not highly expressed in blood cells and are less likely affected by factors other than the disease under investigation. In order to find out which miRNAs detected from plasma are derived from blood cells, miRNA profiling was carried out from pooled whole blood RNA samples at four time-points of the menstrual cycle. The results showed that most miRNAs present on used assays were detected from both sample types, but there were also a number of miRNAs present only in plasma or blood. More miRNAs were detected from blood cells compared to plasma. Most miRNAs detected from both sample types revealed higher expression in whole blood. However, the relative level of several miRNAs was also higher in plasma samples (Table S3) suggesting that some miRNAs in plasma are derived from other tissues and cells besides blood cells. It has been shown that miRNAs originating from tumour tissues can enter the circulation [2]. In addition, miRNAs are delivered to plasma via exosomes or protein complexes, for instance, in pregnant women, placenta-derived exosomes are released to maternal blood [27]. In our study, some miRNAs detected only in plasma samples have been found in other tissues as well. For instance, miR-10b-5p, miR-205-5p and miR-214-3p are shown to be expressed in mouse and human ovaries [28,29]; the expression of miR-572, miR-214-3p and miR-205-5p have been detected in human endometrium [30–32]. Still, the comparison of our and previous studies revealed a large overlap between the results showing that the most highly expressed circulating cell-free miRNAs are miR-451a, miR-486-5p and miR-92a [16,33,34] that are also highly expressed in blood cells [15,16].

miRNAs in whole blood are mainly derived from the mature RBC population [15,35]. In our study, the RBC-derived miRNAs [16] were also the most abundant miRNAs, where miR-451a, miR-144-3p and miR-16-5p showed the highest expression levels in whole blood samples. 

Some limitations of the study should be emphasised. As only a particular set of miRNAs was studied (limited with the presence in Exiqon assays), it is possible that the expression levels of other plasma miRNAs alter during the menstrual cycle. Also, it cannot be ruled out that the lack of significant miRNA expression differences may be caused by the relatively small number of studied samples insufficient to detect small fluctuations in expression levels. In addition, limitations of the data derived from pooled blood samples should be considered as sample pooling results in a loss of information about individual variation. Therefore, miRNAs that were uniquely detected in plasma samples could have been absent from blood pools due to a loss of inter-individual variation as the detection of rare transcripts is more affected by sample pooling [36]. 

In conclusion, we found no differences in plasma miRNA expression levels between the menstrual cycle time-points. This observation can be used for future miRNA biomarker studies for various pathologies including gynaecological diseases. There are circulating cell-free miRNAs in plasma that are not detected or detected at low levels in whole blood samples, still, most miRNAs in plasma are expressed in blood cells as well. 

## Supporting Information

Figure S1 **Cluster analysis of miRNA expression in blood and plasma.** Numbers E10 - E37 represent the study subjects, 1 P- cycle day 1, 2 P- cycle day 7, 3 P- LH day 0, and 4 P- LH day 7. BC- blood samples. Red represents miRNAs with higher expression and green with lower expression compared to the average expression level.
(TIF)

Table S1 **Normalised miRNA expression values (ΔCt) in pooled blood RNA samples.**
(XLS)

Table S2 **A list of miRNAs detected from blood and plasma samples.**
(XLS)

Table S3 **miRNAs with significantly different relative expression values between plasma and blood.**
(XLS)
