# Supplementary figures and images for: Circulating microRNA Profile throughout the Menstrual Cycle
Source: PLoS One. 2013 Nov 14;8(11):e81166. doi: 10.1371/journal.pone.0081166 (PMC3828277; doi:10.1371/journal.pone.0081166)

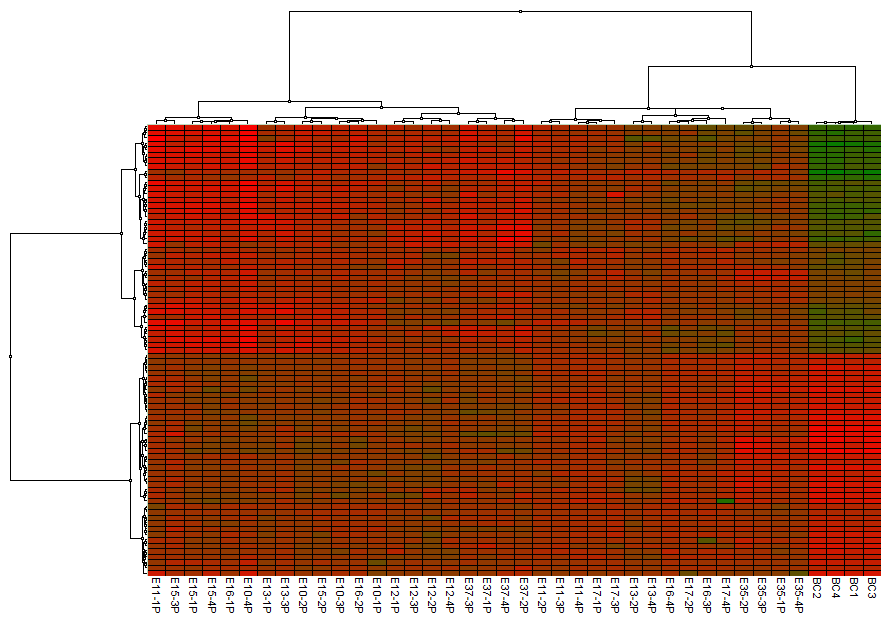

Supplement: Figure S1 — Cluster analysis of miRNA expression in blood and plasma. Numbers E10 - E37 represent the study subjects, 1 P- cycle day 1, 2 P- cycle day 7, 3 P- LH day 0, and 4 P- LH day 7. BC- blood samples. Red represents miRNAs with higher expression and green with lower expression compared to the average expression level. (TIF) [file pone.0081166.s001.tif]
